# Natural Hosts and Genetic Diversity of the Emerging Tomato Leaf Curl New Delhi Virus in Spain

**DOI:** 10.3389/fmicb.2019.00140

**Published:** 2019-02-20

**Authors:** Miguel Juárez, María Pilar Rabadán, Luis Díaz Martínez, Monia Tayahi, Ana Grande-Pérez, Pedro Gómez

**Affiliations:** ^1^Escuela Politécnica Superior de Orihuela, Universidad Miguel Hernández de Elche, Alicante, Spain; ^2^Centro de Edafología y Biología Aplicada del Segura – Consejo Superior de Investigaciones Científicas (CEBAS-CSIC), Departamento Biología del Estrés y Patología Vegetal, Murcia, Spain; ^3^Instituto de Hortofruticultura Subtropical y Mediterránea “La Mayora”, Universidad de Málaga, Consejo Superior de Investigaciones Científicas (IHSM-UMA-CSIC), Área de Genética, Facultad de Ciencias, Málaga, Spain; ^4^Laboratory of Molecular Genetics, Immunology and Biotechnology, Faculty of Sciences of Tunis, Tunis El Manar University, Tunis, Tunisia

**Keywords:** begomovirus, genetic diversity, molecular epidemiology, host range, ToLCNDV

## Abstract

Knowledge about the host range and genetic structure of emerging plant viruses provides insights into fundamental ecological and evolutionary processes, and from an applied perspective, facilitates the design and implementation of sustainable disease control measures. Tomato leaf curl New Delhi virus (ToLCNDV) is an emerging whitefly transmitted begomovirus that is rapidly spreading and inciting economically important diseases in cucurbit crops of the Mediterranean basin. Genetic characterization of the ToLCNDV Mediterranean populations has shown that they are monophyletic in cucurbit plants. However, the extent to which other alternative (cultivated and wild) hosts may affect ToLCNDV genetic population structure and virus prevalence remains unknown. In this study a total of 683 samples from 13 cultivated species, and 203 samples from 24 wild species from three major cucurbit-producing areas of Spain (Murcia, Alicante and Castilla-La Mancha) from five cropping seasons (2012–2016) were analyzed for ToLCNDV infection. Except for watermelon, ToLCNDV was detected in all cultivated-cucurbit species as well as in tomato. Among weeds, *Ecballium elaterium, Datura stramonium, Sonchus oleraceus*, and *Solanum nigrum* were identified as alternative ToLCNDV plant hosts, which could act as new potential sources of virus inoculum. Furthermore, we performed full-genome deep-sequencing of 80 ToLCNDV isolates from different hosts, location and cropping year. Our phylogenetic analysis supports a Mediterranean virus population that is genetically very homogeneous, with no clustering pattern, and clearly different from Asian virus populations. Additionally, *D. stramonium* displayed higher levels of within-host genetic diversity than cultivated plants, and this variability appeared to increase with time. These results suggest that the potential ToLCNDV adaptive evolution occurring in wild plant hosts could serve as a source of virus genetic variability, thereby affecting the genetic structure and spatial-temporal dynamics of the viral population.

## Introduction

Emerging infectious diseases among cultivated plant species represents a serious threat to food sustainability (Woolhouse et al., 2005; Cleaveland et al., 2007; Jones, 2009). These diseases are described as the appearance of an unknown pathogen in a particular plant species, or an already-known pathogen that increases its incidence or arises in a new geographical area often accompanied by drastic reductions in both fruit quality and yield, usually as a consequence of the lack of effective countermeasures (Woolhouse and Gowtage-Sequeria, 2005; Woolhouse et al., 2005; Cleaveland et al., 2007; Woolhouse, 2008; Vurro et al., 2010). Among them, whitefly transmitted virus diseases have been constantly emerging and are a major concern for agriculture (Navas-Castillo et al., 2011, 2014). As an example, outbreaks of tomato leaf curl New Delhi virus (ToLCNDV; genus *Begomovirus*, family *Geminiviridae*) have recently emerged in the cucurbit crops of the Mediterranean basin (Spain, Italy, Tunisia and Morocco), indeed compromising cucurbit productivity and quality (Juárez et al., 2014; Mnari-Hattab et al., 2015; Fortes et al., 2016; Panno et al., 2016; Yazdani-Khameneh et al., 2016; Parrella et al., 2017; Sifres et al., 2017; Zaidi et al., 2017). However, despite the efforts conducted for the control of ToLCNDV diseases, no resistant cultivars to ToLCNDV infection have been identified yet (Sáez et al., 2016). Hence, it is necessary to determine the range of natural hosts and to examine the genetic diversity and structure of ToLCNDV populations, as this knowledge could help improve the design of an integrated disease management program to reduce the source of the inoculum and the spread of avoid.

Tomato leaf curl New Delhi virus is a bipartite begomovirus with two circular ssDNA genome components (DNA-A and DNA-B) of approximately 2.5–2.7 kb (Padidam et al., 1995; Brown et al., 2015; Zaidi et al., 2017). This virus is limited to the plant phloem and is transmitted in a circulative persistent manner by the whitefly *Bemisia tabaci* (Fondong, 2013; Zaidi et al., 2017), although it should be noted that experimental host plants have also been infected by mechanical inoculation (Ruiz et al., 2017). ToLCNDV has a wide host range with 43 different plant species identified thus far (Pratap et al., 2011; Sayed et al., 2013; Kushwaha et al., 2015; Yazdani-Khameneh et al., 2016; Zaidi et al., 2016, 2017; Figàs et al., 2017; Ruiz et al., 2017), mainly belonging to the *Solanaceae* and *Cucurbitaceae* families, which comprise economically important crops such as potato (Usharani et al., 2004) and aubergine (Pratap et al., 2011), as well as to several weed species. However, since the emergence of ToLCNDV in some Mediterranean countries, it has only been described to infect cucurbit and tomato plants (Juárez et al., 2014; Mnari-Hattab et al., 2015; Fortes et al., 2016; Panno et al., 2016; Yazdani-Khameneh et al., 2016; Sifres et al., 2017; Zaidi et al., 2017), while other plant hosts can also be involved in the distribution and prevalence of this virus (Ruiz et al., 2017). Molecular characterization of the ToLCNDV populations has displayed a geographical structure between the ToLCNDV isolates that are currently circulating in Asian and European countries (Zaidi et al., 2017). And in particular, from the first identification of ToLCNDV in Murcia (Spain), the genetic diversity of ToLCNDV populations in Spain has been determined on zucchini and melon plants, based on restriction fragment length polymorphism analysis (RFLP), showing a genetically homogeneous ToLCNDV population composed of isolates belonging to the new ToLCNDV-ES genotype (Fortes et al., 2016). However, whether new ToLCNDV genotypes introduction events have occurred since its emergence in Spain or whether different variants of this virus are associated with specific host species remains unknown, and this could be shaping the evolutionary dynamics and epidemiology of this viral disease in cucurbit crops.

Thus, the natural host range of ToLCNDV from cultivated and wild species collected in three major cucurbit-producing areas of Spain (Murcia, Alicante and Castilla-La Mancha) from 2012 to 2016 was studied in the present work. The genetic variability and population structure of ToLCNDV were examined by phylogenetic and population genetic analyses using full-length genomes obtained from the next-generation sequencing (NGS) data. This study provides fundamental information for identifying alternative host plant species of ToLCNDV, also examining the evolutionary relationships and intra-host genetic diversity of ToLCNDV isolates from Spain. The results could help to elucidate the epidemiological basis of the prevalence and expansion of ToLCNDV in crops.

## Materials and Methods

### Sample Collection

A total of 886 apical leaf samples from cucurbit plants (683) showing virus-like symptoms of ToLCNDV infection and wild plants (203) were collected from (and close to) cucurbit crops grown in greenhouses or open fields (Table 1). These samples extended to 13 plant cultivated species belonging to 5 families: *Cucurbitaceae, Solanaceae, Asteraceae, Apiaceae*, and *Fabaceae*, and 24 wild plant species belonging to 13 families: *Cucurbitaceae, Solanaceae, Asteraceae, Amarantaceae, Boraginacea, Brassicaceae, Chenopodiaceae, Convolvulaceae, Geraniaceae, Malvaceae, Portulacaceae, Urticaceae*, and *Poaceae*, which were collected in South-Eastern Spain (Murcia) for five consecutive cropping seasons (2012–2016), and additional plant samples from Alicante and Castilla La-Mancha were included for the last years studied (Table 1).

**Table 1:** ToLCNDV detection by ELISA and tissue-print hybridization test for cultivated and wild plant samples collected in the major producing-areas in Spain for the 5 consecutive year survey (2012–2016).

	Family	Species	N° analyzed samples (N° positive samples)
**Cultivated plant species**	*Cucurbitaceae*	Melon	197 (157)
	*Cucurbitaceae*	Zucchini	191 (182)
	*Cucurbitaceae*	Pumpkin	36 (23)
	*Cucurbitaceae*	Cucumber	36 (18)
	*Cucurbitaceae*	Watermelon	43 (0)
	*Solanaceae*	Tomato	46 (7)
	*Solanaceae*	Eggplant	37 (0)
	*Solanaceae*	Potato	16 (0)
	*Solanaceae*	Pepper	47 (0)
	*Asteraceae*	Lettuce	7 (0)
	*Apiaceae*	Celery	6 (0)
	*Fabaceae*	Bean	9 (0)
	*Fabaceae*	Broad bean	12 (0)
	*TOTAL*		683 (387)
**Wild plant species**	*Cucurbitaceae*	*Ecballium elaterium*	18 (16)
	*Asteraceae*	*Sonchus oleraceus*	16(2)
	*Solanaceae*	*Datura stramonium*	6 (4)
	*Solanaceae*	*Solanum nigrum*	11 (4)
	*Solanaceae*	*Nicotiana glauca*	14(0)
	*Amarantaceae*	*Amaranthus blitoides*	18 (0)
	*Amarantaceae*	*Amaranthus retroflexu*s	6 (0)
	*Asteraceae*	*Chrysanthemum coronarium*	6 (0)
	*Asteraceae*	*Senecio vulgari*s	8 (0)
	*Asteraceae*	*Conyza bonariensis*	7 (0)
	*Boraginaceae*	*Echium plantagineum*	5 (0)
	*Boraginaceae*	*Heliotropium europaeum*	12 (0)
	*Brasicaceae*	*Cardaria draba*	9 (0)
	*Brasicaceae*	*Moricandia arvensis*	5 (0)
	*Brasicaceae*	*Sinapis arvensis*	5 (0)
	*Quenopodiaceae*	*Chenopodium album*	12 (0)
	*Quenopodiaceae*	*Chenopodium murale*	10 (0)
	*Convolvulaceae*	*Convolvulus arvensi*s	3 (0)
	*Geraniaceae*	*Erodium chium*	3 (0)
	*Malvaceae*	*Abutilon theophrasti*	8 (0)
	*Malvaceae*	*Malva spp.*	8 (0)
	*Portulacaceae*	*Portulaca oleracea*	4 (0)
	*Urticaceae*	*Urtica urens*	6 (0)
	*Poaceae*	*Setaria viridis*	3 (0)
	*TOTAL*		203 (26)


### ToLCNDV Detection

Plant samples were double-screened for ToLCNDV infection by DAS-ELISA using IgG (AS-1109, DSMZ) and according to the common procedure (Clark and Adams, 1977), as well as by non-isotopic tissue-printing hybridization analysis (Más and Pallás, 1995). Molecular hybridization was carried out from plant leaf petioles, which were cut transversely and then printed twice onto a positively charged nylon membrane (Amersham Pharmacia Biotech), including samples of healthy plants as negative controls. The membrane was irradiated with UV light in a cross-linker, and then DNA:DNA hybridization detection was carried out using a specific DNA probe kindly provided by D. Janssen (IFAPA-La Mojonera, Almería, Spain). Briefly, a DNA sequence corresponding to 351-bp segments of the coat protein gene (DNA-A) from the ToLCNDV-ES (KT175406) isolate was cloned into pGEM-T-easy vector following the manufacturer’s instructions, and used to synthesize the specific DNA probe labeled with digoxigenin by PCR (Sambrook and Russell, 2001). The molecular hybridization procedure was carried out as previously described (Alfaro-Fernández et al., 2016) with 2 h of pre-hybridization and 42°C of o/n incubation with the digoxigenin-labeled probe.

### Next-Generation Sequencing and Bioinformatics Analysis

We used a random collection of 80 samples (Supplementary Table S1) based on their location, host and year, and which had tested positive for ToLCNDV infection. Total DNA was extracted from each plant using the cetyltrimethylammonium bromide (CTAB)-based procedure (Doyle et al., 1990) and stored at –20°C until use. For each sample, total DNA (10 ng/μl) was used to amplify the ToLCNDV genome by rolling circle amplification (RCA) using ϕ29 DNA polymerase (TempliPhi kit, GE Healthcare, Little Chalfont, United Kingdom) (Inoue-Nagata et al., 2004; Haible et al., 2006). All samples generated amplification products that were digested by the restriction enzymes *Noc* I and *Bstx* I that cut at a unique restriction site for DNA-A and DNA-B, respectively, confirming that all the products were linearized to similar size fragments by electrophoresis in a 1% agarose gel (Juárez et al., 2014). The eighty RCA products generated from circular DNA were purified with AMPure XP beads, and used for the Illumina library preparation that was carried out using a Nextera XT library kit with the subsequent run on the Illumina MiSeq platform (2 × 300 bp length paired-end reads). Adapters and low-quality sequences from the NGS data (below QC26) and other contaminant reads were removed by using SeqTrim next software (Falgueras et al., 2010). The total number of mapped nucleotides ranged from 51,468,017 to 316,919, thereby generating an amount of viral reads that varied from 263,427 to 2,521, respectively (Supplementary Table S2). ToLCNDV reads were aligned against ToLCNDV reference genomes A and B (isolate Murcia 8.1 GenBank accession numbers KF749224.1 and KF749227.1, respectively), and all consensus sequences had a percentage identity >99.9%. Consensus sequences of genomes A and B were obtained with SeqmanNGen 14. DNA extracts coming from the zucchini plants with severe symptoms collected in Murcia in 2016 were tested for the presence of betasatellite DNA by PCR, using the improved universal primers CLB36F and CLB37R reported to detect a vast range of betasatellites associated with begomoviruses across a range of crops (Kumar et al., 2018). In order to detect the presence of other circular DNA, such as virus or betasatellites, NGS data were also subjected to *de novo* assembly using SeqmanNGen 14.

### Phylogenetic and Population Structure Analysis

The phylogenetic relationships between ToLCNDV isolates were inferred from a collection of 50 full-genome ToLCNDV sequences retrieved from GenBank (and referenced in this study according to their accession number), including the Spanish full-length isolate sequences for genome A (KF749224; KF891468; KF749223; KM977733; and KF749225) and B (KF891467; KM977734; KF749228; KF749227; and KF749226) described in Juárez et al. (2014) and Ruiz et al. (2014), and the 80 full-genome sequences that were determined in this work. All 130 full-genome sequences were separated into genome A and B groups, and aligned by using the Multiple Sequence Comparison by Log-Expectation (MUSCLE). The evolutionary history was inferred by using the Maximum Likelihood method based on the Tamura-Nei model performed in MEGA7 (Kumar et al., 2016). Initial tree(s) for the heuristic search were automatically obtained by applying the Neighbor-Joining and BioNJ algorithms to a matrix of pairwise distances estimated using the Maximum Composite Likelihood (MCL) approach, and then by selecting the topology with superior log likelihood value. Analysis of molecular variation (AMOVA) was carried out in R with the Poppr package (Kamvar et al., 2015) and was conducted to estimate the portion of molecular variation into potential population differentiation due to variation within- and between-sub-populations partitioned by host, year and location.

### Mutant-Swarm Analysis Within-Host

Based on the read depth (1000x) and quality filters (QC26), a comprehensive collection of 46 ToLCNDV samples from NGS data was assembled in order to estimate genetic parameters that reflected the mutant spectra of each sample (Table 2). SNPs and InDels called by VarScan2 (Koboldt et al., 2012a,b) were filtered and only the ones occurring ten or more times were considered reliable. Within each mutant spectrum, mutations were counted against the consensus sequence. Mutational frequency (mut/nt) was calculated by scoring the number of different mutations divided by the total number of nucleotides sequenced (Sánchez-Campos et al., 2018), while mutational frequency for each gene (mut. freq/codon) was estimated as the addition of the mutation frequencies per codon of all codons in the gene divided by the total of codons. Mutant spectra heterogeneity was estimated by using the normalized Shannon entropy (Sánchez-Campos et al., 2018). Finally, the haplotype reconstruction, as the inference of the group of variants that was assembled in the full-length sequence, was performed with the software Haploclique (Topfer et al., 2014). The statistical significance of the intra-swarm variability among samples was carried out via a two-way ANOVA fitting the host, year and location as factors and their interactions as appropriate. As for the number of SNPs, an analysis was carried out by using generalized linear models (GLM) with a Poisson error distribution. All statistical analyses were performed using R software.

**Table 2:** Estimation of the within-population genetic parameters for genome A of the ToLCNDV isolates. Samples are listed according to the location, year and host, and values represent the mutation frequency (mutations/nucleotide position) for each genome, while mutation frequency for each gene was estimated as the mutation frequency per codon. Numbers of SNPs, indels, nucleotide diversity and Shannon index were estimated by retracting the 10^-3^ error rate correction.

Sample	Place	Year	Plant	Mutation frequency	Mutation frequency per codon						SNPs	Indels	Nuc.Div_Entity	Shannon index
									
					AV2	CP	AC4	Ren	TrAp	Rep				
**A-MU.12.ZU/1.1**	Murcia	2012	Zucchini	1.20E-03	1.40E-03	1.00E-03	1.40E-03	8.00E-04	3.70E-03	1.00E-03	0	0	0	0
**A-MU.12.ZU/3.1**	Murcia	2012	Zucchini	1.20E-03	1.00E-03	8.00E-04	1.10E-03	1.50E-03	9.00E-04	4.00E-04	0	0	0	0
**A-MU.12.ZU/4.1**	Murcia	2012	Zucchini	1.10E-03	1.60E-03	9.00E-04	5.70E-03	9.00E-04	6.60E-03	1.79E-02	17	1	2.10E-03	5.41E-01
**A-MU.12.ZU/6.1**	Murcia	2012	Zucchini	1.10E-03	1.00E-03	1.00E-03	1.27E-02	5.30E-03	1.75E-02	1.78E-02	21	4	2.40E-03	7.37E-01
**A-MU.13.ZU/7.1**	Murcia	2013	Zucchini	1.20E-03	8.00E-04	8.00E-04	8.00E-04	8.00E-04	1.11E-02	1.77E-02	12	1	1.80E-03	8.08E-01
**A-MU.13.ZU/8.1**	Murcia	2013	Zucchini	1.10E-03	8.00E-04	9.00E-04	1.10E-03	1.30E-03	9.30E-03	9.00E-04	19	2	3.10E-03	8.05E-01
**A-MU.13.ZU/9.1**	Murcia	2013	Zucchini	1.80E-03	1.10E-03	2.20E-03	2.23E-02	8.00E-04	1.72E-02	1.77E-02	3	0	6.00E-04	7.19E-01
**A-MU.14.ZU/15.1**	Murcia	2014	Zucchini	1.10E-03	8.00E-04	9.00E-04	1.10E-03	9.00E-04	3.70E-03	7.00E-04	2	1	4.00E-04	9.99E-01
**A-MU.14.ZU/16.1**	Murcia	2014	Zucchini	1.30E-03	1.30E-03	8.00E-04	2.30E-02	8.30E-03	9.60E-03	1.77E-02	22	3	2.10E-03	8.27E-01
**A-AL.14.ZU/17.1**	Alicante	2014	Zucchini	1.00E-03	9.00E-04	1.10E-03	8.10E-03	8.00E-04	1.47E-02	1.76E-02	11	1	3.20E-03	6.13E-01
**A-MU.14.ZU/18.1**	Murcia	2014	Zucchini	1.00E-03	8.00E-04	9.00E-04	1.55E-02	8.20E-03	1.75E-02	7.00E-04	3	1	7.00E-04	9.89E-01
**A-MU.15.ME/19.1**	Murcia	2015	Melon	1.20E-03	1.10E-03	1.10E-03	1.60E-03	1.20E-03	4.40E-03	1.10E-03	8	2	1.00E-03	9.93E-01
**A-AL.15.ME/21.1**	Alicante	2015	Melon	1.50E-03	4.70E-03	2.70E-03	1.50E-03	1.60E-03	4.20E-03	1.00E-03	5	0	9.00E-04	8.43E-01
**A-MU.15.CU/22.1**	Murcia	2015	Cucumber	1.30E-03	1.30E-03	1.10E-03	1.60E-03	8.80E-03	5.40E-03	1.30E-03	0	0	0	0
**A-MU.15.CU/23.1**	Murcia	2015	Cucumber	1.20E-03	1.20E-03	1.20E-03	1.59E-02	1.10E-03	7.00E-03	1.78E-02	24	2	2.10E-03	6.04E-01
**A-AL.15.ZU/25.1**	Alicante	2015	Zucchini	9.00E-04	8.00E-04	1.00E-03	9.00E-04	9.00E-04	3.80E-03	1.30E-03	0	1	0	0
**A-MU.15.PU/26.1**	Murcia	2015	Pumpkin	1.30E-03	7.00E-04	1.20E-03	1.00E-03	7.00E-04	8.30E-03	1.93E-02	0	0	0	0
**A-MU.15.PU/27.1**	Alicante	2015	Pumpkin	1.30E-03	9.60E-03	1.00E-03	1.54E-02	9.00E-04	6.80E-03	1.30E-03	3	1	1.20E-03	9.25E-01
**A-MU.16.DA/12.1**	Murcia	2016	Datura	2.60E-03	3.10E-03	7.30E-03	4.90E-03	3.70E-03	1.29E-02	3.30E-03	0	0	0	0
**A-MU.16.ZU/44.1**	Murcia	2016	Zucchini	3.10E-03	3.50E-03	4.00E-03	1.19E-02	1.09E-02	1.79E-02	2.04E-02	0	0	0	0
**A-MU.16.ZU/40.1**	Murcia	2016	Zucchini	2.90E-03	4.50E-03	4.70E-03	4.50E-03	4.10E-03	1.50E-02	3.90E-03	0	0	0	0
**A-MU.16.DA/17.1**	Murcia	2016	Datura	3.50E-03	3.40E-03	4.10E-03	1.37E-02	5.50E-03	1.11E-02	2.06E-02	29	1	1.50E-03	9.14E-01
**A-MU.16.DA/18.1**	Murcia	2016	Datura	4.00E-03	4.10E-03	4.50E-03	1.98E-02	7.80E-03	1.78E-02	3.50E-03	14	1	1.70E-03	8.12E-01
**A-MU.16.ME/19.1**	Murcia	2016	Melon	2.50E-03	1.21E-02	4.10E-03	4.80E-03	4.00E-03	2.06E-02	3.20E-03	5	0	7.00E-04	6.34E-01
**A-MU.16.ME/21.1**	Murcia	2016	Melon	2.80E-03	3.50E-03	4.10E-03	1.20E-02	1.14E-02	1.00E-02	3.70E-03	7	1	1.80E-03	9.34E-01
**A-MU.16.PU/23.1**	Alicante	2016	Pumpkin	2.70E-03	3.70E-03	4.00E-03	5.00E-03	4.80E-03	6.90E-03	4.00E-03	0	0	0	0
**A-AL.16.ME/24.1**	Alicante	2016	Melon	2.90E-03	4.00E-03	4.60E-03	5.20E-03	4.50E-03	7.50E-03	4.40E-03	0	0	0	0
**A-AL.16.ZU/27.1**	Alicante	2016	Zucchini	2.60E-03	2.60E-03	3.60E-03	3.80E-03	3.30E-03	3.70E-03	3.00E-03	0	0	0	0
**A-AL.16.ME/28.1**	Alicante	2016	Melon	3.20E-03	4.40E-03	4.10E-03	1.71E-02	4.70E-03	1.12E-02	9.00E-03	1	1	0	0
**A-AL.16.ZU/32.1**	Alicante	2016	Zucchini	5.00E-03	3.40E-03	4.30E-03	1.60E-02	1.42E-02	1.58E-02	1.12E-02	0	0	0	0
**A-MU.16.CU/34.1**	Murcia	2016	Cucumber	3.60E-03	3.80E-03	5.10E-03	4.80E-03	4.30E-03	1.06E-02	3.70E-03	0	0	0	0
**A-AL.16.CU/35.1**	Alicante	2016	Cucumber	3.00E-03	3.50E-03	4.50E-03	1.21E-02	4.00E-03	1.59E-02	3.50E-03	27	0	1.50E-03	7.69E-01
**A-MU.16.PU/37.1**	Alicante	2016	Pumpkin	2.80E-03	3.30E-03	3.90E-03	5.10E-03	3.70E-03	9.40E-03	3.50E-03	0	2	0	0
**A-MU.16.PU/38.1**	Alicante	2016	Pumpkin	3.00E-03	3.40E-03	4.20E-03	5.10E-03	3.80E-03	1.23E-02	2.06E-02	4	0	4.30E-03	9.15E-01
**A-MU.16.ZU/13.1**	Murcia	2016	Zucchini	4.40E-03	3.40E-03	4.00E-03	1.15E-02	3.30E-03	2.01E-02	2.00E-02	88	1	2.30E-03	8.99E-01
**A-MU.16.ZU/14.1**	Murcia	2016	Zucchini	2.50E-03	3.30E-03	3.80E-03	1.97E-02	4.00E-03	6.80E-03	3.80E-03	6	0	2.40E-03	8.54E-01
**A-MU.16.ZU/15.1**	Murcia	2016	Zucchini	2.80E-03	3.20E-03	4.10E-03	1.29E-02	4.00E-03	1.51E-02	1.99E-02	2	0	1.70E-03	7.53E-01
**A-MU.16.ZU/16.1**	Murcia	2016	Zucchini	3.10E-03	3.70E-03	2.30E-03	1.18E-02	1.21E-02	8.50E-03	2.80E-03	0	0	0	0
**A-LCM.16.ME/4.1**	La Mancha	2016	Melon	2.90E-03	3.20E-03	5.20E-03	6.70E-03	5.80E-03	4.40E-03	3.40E-03	0	0	0	0
**A-LCM.16.ME/5.1**	La Mancha	2016	Melon	2.80E-03	3.60E-03	4.10E-03	5.00E-03	4.30E-03	1.30E-02	2.08E-02	6	0	8.00E-04	7.97E-01
**A-LCM.16.ME/6.1**	La Mancha	2016	Melon	2.90E-03	3.80E-03	4.10E-03	5.80E-03	5.20E-03	1.40E-02	3.40E-03	9	0	9.00E-04	8.23E-01
**A-LCM.16.ME/1.1**	La Mancha	2016	Melon	2.30E-03	1.00E-03	3.00E-03	5.00E-03	3.50E-03	5.40E-03	3.90E-03	0	0	0	0
**A-LCM.16.ME/2.1**	La Mancha	2016	Melon	3.00E-03	3.40E-03	4.00E-03	4.80E-03	3.90E-03	9.20E-03	3.70E-03	0	0	0	0
**A-LCM.16.ME/3.1**	La Mancha	2016	Melon	2.80E-03	3.20E-03	4.00E-03	5.60E-03	4.20E-03	6.50E-03	3.60E-03	5	0	2.60E-03	9.62E-01
**A-MU.16.ZU/46.1**	Murcia	2016	Zucchini	3.20E-03	2.40E-03	3.90E-03	1.05E-02	7.80E-03	6.10E-03	4.86E-02	7	0	2.00E-03	8.43E-01
**A-MU.16.ZU/47.1**	Murcia	2016	Zucchini	3.00E-03	2.90E-03	5.10E-03	1.49E-02	1.09E-02	1.22E-02	3.91E-02	6	2	1.30E-03	8.01E-01


### Data Availability

All sequencing information and data that support the findings of this study have been deposited in the European Nucleotide Archive (ENA) database with the accession code PJRNA479563. Additionally, the complete set of 80 ToLCNDV genome sequences generated in this work were deposited in GenBank under accession numbers MH577603–MH577762.

## Results

### ToLCNDV Detection in Cultivated and Wild Plants

**FIGURE 1:**
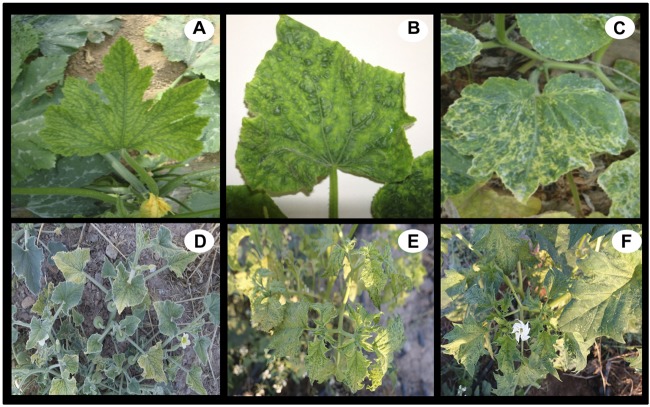
Plant images showing virus-like symptoms of tomato leaf curl New Delhi virus (ToLCNDV) infection in cultivated and wild plant species. The top row shows symptomatic cultivated Zuchinni **(A)**, Cucumber **(B)**, and Melon **(C)** plants, where common symptoms include curling, yellowing and severe mottle mosaic in young leaves with plants that appear stunted in growth. The bottom row shows symptomatic wild *Ecballium elaterium* **(D)**, *Datura stramonium* **(E)**, and *Solanum nigrum* **(F)** plants, comprising curling leaves, chlorotic mottling and mosaic in young leaves, including yellow spots and pale green tissues.

From the first identification of ToLCNDV in cucurbit crops in Spain in 2012, several outbreaks have emerged in cucurbit-producing areas, which have been mainly distributed over greenhouses and open-fields in the South, Central and East areas of Spain. We thus sampled cucurbit plants showing virus-like symptoms of ToLCNDV infection, including other plants near major crops. ToLCNDV symptoms are dependent on the cultivar and growing conditions, although common symptoms may include curling and yellowing in young leaves of zucchini plants, vein swelling in cucumber plants, and severe mosaic in melon plants. In addition, these plants appear stunted in growth (Figure 1A–C). A total of 683 apical leaf samples were collected from 13 cultivated plant species belonging to 5 different families (Table 1). Except for watermelon plants, ToLCNDV was detected in most samples from cultivated cucurbit species (melon, zucchini, cucumber and pumpkin). Surprisingly, despite the watermelon plant surveys being conducted in crop fields close to other cucurbit crops with high whitefly populations, that is, under high natural inoculum pressure, ToLCNDV was not detected in any watermelon samples. More interestingly, fifteen out of 43 samples came from watermelon plants that had been grafted onto the pumpkin hybrid rootstock, and while their sprouts on the base-stem showed a strong ToLCNDV symptomatology, all watermelon samples were negative for ToLCNDV. Within *Solanaceae* crop species, there were only 7 tomato samples (out of 46) that were positive for ToLCNDV, while ToLCNDV was unnoticed neither in aubergine (*Solanum melongena*), pepper (*Capsicum annuum*), potato (*Solanum tuberosum*), nor in lettuce (*Lactuca sativa*), celery (*Apium graveolens*), bean (*Phaseolus vulgaris*) and broad bean (*Vicia faba*) plants. Note that there were no differences between the test results performed by DAS-ELISA and hybridization.

Among weed plants, a total of 203 apical leaf samples were collected from 24 wild species belonging to 13 different families (Table 1). The sampling was based on weeds growing adjacent to melon and zucchini fields in Murcia, either being asymptomatic or showing virus-like symptoms. ToLCNDV was detected in 4 wild species; *Ecballium elaterium, Datura stramonium, Sonchus oleraceus*, and *Solanum nigrum*. In particular, the ToLCNDV occurrence in *Ecballium, Datura*, and *Sonchus* weed plants is described here for the first time and they could be considered as potential alternative hosts. More specifically, the frequency of ToLCNDV detection in *E. elaterium* and *D. stramonium* was high (66–88%), although the extensive presence of *D. stramonium* over cucurbit fields colonized by *B. tabaci* suggested that this host could play an important role as a potential source of ToLCNDV inoculum in cucurbit crops. As for the symptoms that could reveal the possible presence of ToLCNDV, *E. elaterium* exhibited yellowing with sharply bordered yellow leaves, *D. stramonium* showed malformations with pale yellow and green spots in young leaves, and *S. nigrum* presented chlorotic spots in young leaves (Figure 1D–F).

### Genetic Structure of the ToLCNDV Population

**FIGURE 2:**
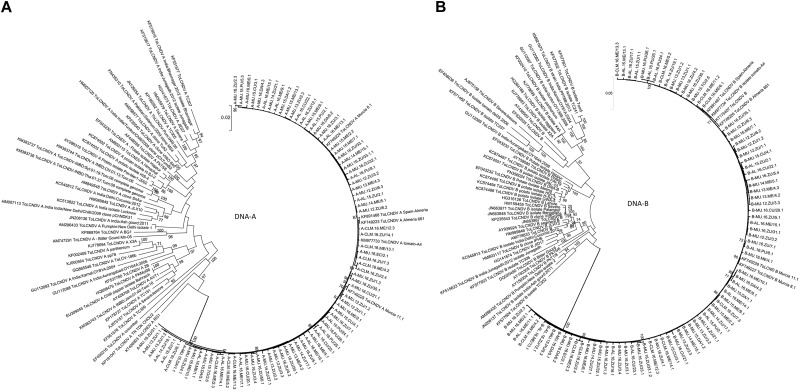
Phylogenetic trees of the 80 ToLCNDV consensus nucleotide sequences. The phylogenetic analysis was inferred by Maximum Likelihood, using the full-length genome **A** (left) and **B** (right) of the ToLCNDV isolates of this study, including a collection of 50 full-genome ToLCNDV sequences retrieved from NCBI-GenBank (referenced according to their accession number), with also full-length ToLCNDV sequences from Spain available to date. Each ToLCNDV isolate from this study was referenced according to its location (MU, Murcia; AL, Alicante; CLM, Castilla-La Mancha), year (2012–2016), host (ZU, Zucchini; ME, Melon; PU, Pumpkin; CU, Cucumber; DA, *D. stramonium*), as well as the plot and sample number. The bootstrap values corresponded to 1,000 replications, and the scales provide genetic distances.

The genetic population structure of ToLCNDV was examined by phylogenetic and population genetic analyses using full-length genomes from a random collection of 80 isolates (Supplementary Table S1) grouped by location, host and year. The phylogenetic relationship between the 80 full-genome ToLCNDV sequences from this study and the 50 full-genome sequences retrieved from NCBI-GenBank showed a very genetically homogeneous Spanish virus population, with no clustering pattern, and quite differentiated from those ToLCNDV isolates reported from Indian subcontinent (Figure 2). Similar results were obtained using the Neighbor-Joining and Maximum Likelihood algorithms. This uniform clade within the Spanish ToLCNDV population showing a relatively short genetic distance among isolates (based on the branch length) was consistent with a recent study in which the ToLCNDV population was characterized with an RFLP approach, but only using cucurbit plants (Fortes et al., 2016). Furthermore, we used the collection of the ToLCNDV sequences of this study in order to perform AMOVA analysis to estimate any genetic differentiation according to the area, year and host population. Our results showed that 94–99% of the variation was attributed to differences within Spanish populations (*p* < 0.001), with a scarce portion (2–6%) of the total molecular variation that resulted from differences between populations, indicating that ToLCNDV Spanish populations grouped by host, year and location remained clearly undifferentiated among them. This was consistent with the low levels of genetic diversity, displaying a uniform ToLCNDV population that likely could be associated to the whitefly transmission mode and its high migration rate between different geographical cucurbit-producing areas. In turn, since there was a high nucleotide similarity among these Spanish ToLCNDV isolates, it is possible that this viral population came from a single introduction of a ToLCNDV isolate into Spain (i.e., founder effect), which then became adapted to cucurbit plants. In that respect, a substantial within-host genetic variability consistent with host adaptation could be expected.

### Within-Population Genetic Diversity of ToLCNDV

**FIGURE 3:**
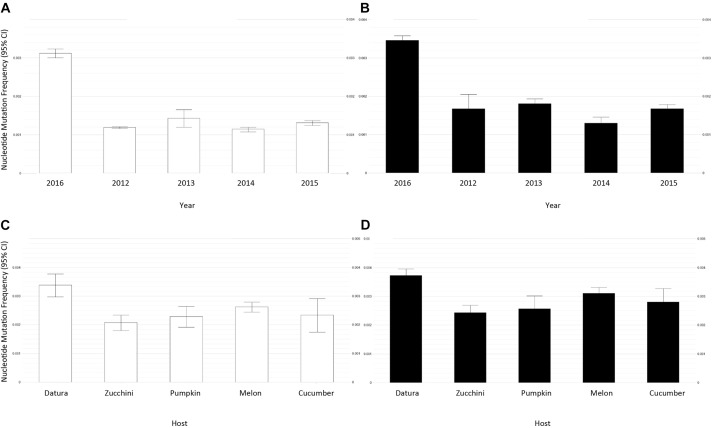
Inference of the heterogeneity within-population of ToLCNDV averaged by the mutation frequency (mutations based on nucleotide substitutions and indels per nucleotide) that was estimated for each viral genomic component **A** (left, white-bars) and **B** (right, black-bars), grouping ToLCNDV populations by collection year (**A,B**; 2012 to 2016), and also by infected host (**C,D**; Cucumber, Melon, Pumpkin, Zucchini, and *D. stramonium*). Bars represent the mean value with the 95% CI error bar.

**FIGURE 4:**
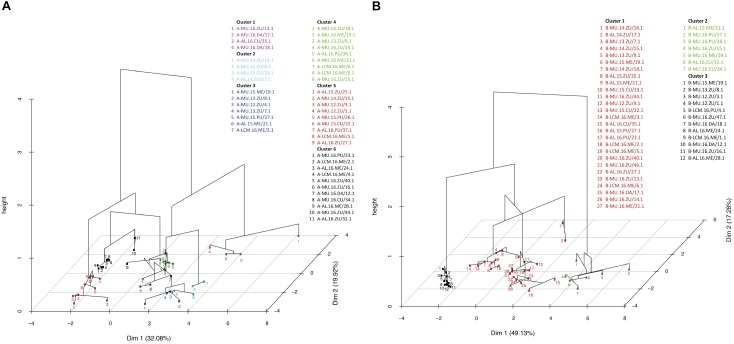
Principal Component Analysis (PCAs) of both genomes for the ToLCNDV isolates. All isolates were clustered based on the correlation matrix of the genetic variables after normalization data. ToLCNDV isolates are colored according to the cluster, and each isolate is named after its location (MU, Murcia; AL, Alicante; CLM, Castilla-La Mancha), year (2012–2016), and host (ZU, Zucchini; ME, Melon; PU, Pumpkin; CU, Cucumber; DA, *D. stramonium*). Supplementary Tables S3 and S4 show the significances and variable responsible for the group separation. In genome **A** (left), the six groups consisted of isolates that originated from the same year, whereas the three groups that originated in genome **B** (right) were fitted according to time and host.

A collection of 46 ToLCNDV samples were assembled from NGS data in order to examine the mutant swarm within-population, as well as to which extent time, host and geographical location affect ToLCNDV genetic variation. Our bioinformatics analysis revealed an average mutational frequency of 6.5 × 10^-3^ to 5.7 × 10^-3^ mutations/nucleotide site for genomes A and B (Tables 2 and 3, respectively). In particular for genome A, the *TrAp, CP*, and *AV2* genes had a higher influence than the rest, exhibiting a significant increase according to time (Table 2 and Figure 3A) (ANOVA: main year effect; *TrAp, F*_1,4_ = 2.88, *p* = 0.04; *CP, F*_1,4_ = 65.71, *p* < 0.001 and *AV2, F*_1,4_ = 16.71, *p* < 0.001). Additionally, the *AV2* genomic region also had an effect on that mutational frequency and was dependent on the host (Figure 3A,C; interaction effects of year and host, *F*_1,3_ = 5.16, *p* < 0.006). *D. stramonium* showed the highest values in the mutation frequency rate (Figure 3C). For genome B, the time factor had a similar effect (Table 3 and Figure 3B), as both *MP* and *NSP* genes also had an influence on the mutation frequency (Figure 3B; main year effect; *MP, F*_1,4_ = 9.76, *p* < 0.001 and *NSP, F*_1,4_ = 14.12, *p* < 0.001). Similarly, the average number of SNPs showed significant differences for genome A in hosts [likelihood-ratio test (LRT) = 105.33, *p* < 0.001], year (LRT = 11.16, *p* < 0.02) and location (LRT = 99.9, *p* < 0.001) as well as for the genome B in hosts (LRT = 109.26, *p* < 0.001), year (LRT = 96.74, *p* < 0.01) and location (LRT = 108.9, *p* < 0.001), with no interactions between these main factors. For instance, *D. stramonium* plants from Murcia-2016 had a higher number of SNPs in their genomes than the rest of the hosts (Tables 2, 3). This result suggests an active exploration of sequence space that may depend on host species, although further studies are required to confirm this point. Note that the estimation of the normalized Shannon entropy for the full-genome sequenced ranged from 0 to 0.1, informing of a high similarity among the sequences. Next, PCA analysis based on all within-host genetic variables revealed a clear pattern of genotypic similarities among those ToLCNDV populations from the same year and host (Figure 4). The statistic results of the PCA dimension and significance for variables responsible of the separation are summarized in Supplementary Tables S3 and S4 for genome A and B, respectively. In particular, using the genome A data, the regression of the resultant factors was significant for time (Supplementary Table S3, *R*^2^ = 0.68, *p* < 0.001), grouping the isolates into 6 clusters. Likewise, for the genome B, these isolates were grouped into 3 clusters that fitted for time and host plant (Supplementary Table S4; year, *R*^2^ = 0.68 *p* < 0.001; plant, *R*^2^ = 0.20, *p* < 0.001). Overall, these results suggest that despite the relatively low levels of genetic diversity observed from the above consensus data, possibly as a result of genetic drift and population bottlenecks, there is a temporal and host effect that is reflected in the moderate levels of intra-host genetic variation in both ToLCNDV genomes. This could indicate a clear potential adaptive evolution over time, which is further enhanced in and by wild plants, having an important role on the genetic structure and spatiotemporal dynamics of ToLCNDV population in crops.

**Table 3:** Estimation of the within-population genetic parameters for genome B of the ToLCNDV isolates. Samples are listed by fitting the location, year and host, and values show the mutation frequency (mutations/nucleotide position) for each genome, while mutation frequency for each gene was estimated by mutation frequency per codon. Numbers of SNPs, indels, nucleotide diversity and Shannon index were estimated by retracting the 10^-3^ error rate correction.

Sample	Place	Year	Plant	Mutation frequency	Mutation frequency per codon		SNPs	Indels	Nucleotide diversity	Shannon index
									
					NSP	MP				
**B-MU.12.ZU/1.1**	Murcia	2012	Zucchini	1.00E-03	4.60E-03	4.70E-03	0	0	0	0
**B-MU.12.ZU/3.1**	Murcia	2012	Zucchini	1.20E-03	1.00E-03	1.20E-03	0	0	0	0
**B-MU.12.ZU/4.1**	Murcia	2012	Zucchini	2.70E-03	1.10E-03	2.40E-03	0	0	0	0
**B-MU.12.ZU/6.1**	Murcia	2012	Zucchini	1.60E-03	4.80E-03	6.20E-03	10	0	1.20E-03	8.58E-01
**B-MU.13.ZU/7.1**	Murcia	2013	Zucchini	1.50E-03	8.00E-04	9.00E-04	14	0	1.70E-03	6.69E-01
**B-MU.13.ZU/8.1**	Murcia	2013	Zucchini	1.90E-03	4.70E-03	1.10E-03	5	0	7.00E-04	6.68E-01
**B-MU.13.ZU/9.1**	Murcia	2013	Zucchini	1.80E-03	4.80E-03	2.00E-03	21	0	1.40E-03	7.45E-01
**B-MU.14.ZU/15.1**	Murcia	2014	Zucchini	1.00E-03	4.50E-03	4.50E-03	2	0	4.00E-04	9.20E-01
**B-MU.14.ZU/16.1**	Murcia	2014	Zucchini	1.50E-03	9.00E-04	4.40E-03	28	2	1.80E-03	8.47E-01
**B-AL.14.ZU/17.1**	Alicante	2014	Zucchini	1.60E-03	4.60E-03	8.00E-03	36	3	2.20E-03	7.25E-01
**B-MU.14.ZU/18.1**	Murcia	2014	Zucchini	9.00E-04	4.80E-03	4.60E-03	7	1	4.00E-04	1.58E-01
**B-MU.15.ME/19.1**	Murcia	2015	Melon	1.40E-03	1.60E-03	2.00E-03	6	0	0	0
**B-AL.15.ME/21.1**	Alicante	2015	Melon	2.00E-03	9.00E-03	9.00E-03	70	4	4.10E-03	4.61E-01
**B-MU.15.CU/22.1**	Murcia	2015	Cucumber	1.90E-03	1.50E-03	9.50E-03	0	0	0	0
**B-MU.15.CU/23.1**	Murcia	2015	Cucumber	1.90E-03	1.10E-03	4.70E-03	27	1	1.20E-03	8.89E-01
**B-AL.15.ZU/25.1**	Alicante	2015	Zucchini	1.30E-03	4.80E-03	1.40E-03	5	0	7.00E-04	9.99E-01
**B-MU.15.PU/26.1**	Murcia	2015	Pumpkin	1.30E-03	4.60E-03	1.20E-03	10	0	1.10E-03	8.90E-01
**B-MU.15.PU/27.1**	Alicante	2015	Pumpkin	1.60E-03	1.20E-03	8.50E-03	9	0	8.00E-04	7.18E-01
**B-MU.16.DA/12.1**	Murcia	2016	Datura	3.70E-03	8.00E-03	4.40E-03	0	0	0	0
**B-MU.16.ZU/44.1**	Murcia	2016	Zucchini	2.60E-03	4.40E-03	4.80E-03	17	2	8.00E-04	7.52E-01
**B-MU.16.ZU/46.1**	Murcia	2016	Zucchini	3.20E-03	5.30E-03	9.60E-03	6	1	7.00E-04	8.63E-01
**B-MU.16.ZU/47.1**	Murcia	2016	Zucchini	3.30E-03	4.80E-03	5.50E-03	0	0	0	0
**B-MU.16.ZU/40.1**	Murcia	2016	Zucchini	3.10E-03	4.60E-03	6.10E-03	23	0	1.20E-03	8.94E-01
**B-MU.16.DA/17.1**	Murcia	2016	Datura	4.10E-03	8.50E-03	9.40E-03	22	0	8.00E-04	7.77E-01
**B-MU.16.DA/18.1**	Murcia	2016	Datura	3.30E-03	8.30E-03	5.40E-03	0	0	0	0
**B-MU.16.ME/19.1**	Murcia	2016	Melon	3.50E-03	1.89E-02	1.52E-02	33	1	2.20E-03	8.08E-01
**B-MU.16.ME/21.1**	Murcia	2016	Melon	3.30E-03	1.49E-02	1.43E-02	10	1	9.00E-04	8.43E-01
**B-MU.16.PU/23.1**	Alicante	2016	Pumpkin	2.80E-03	4.60E-03	4.90E-03	13	1	9.00E-04	7.28E-01
**B-AL.16.ME/24.1**	Alicante	2016	Melon	3.30E-03	9.00E-03	5.20E-03	0	0	0	0
**B-AL.16.ZU/27.1**	Alicante	2016	Zucchini	2.80E-03	8.82E-03	4.20E-03	19	0	9.00E-04	7.72E-01
**B-AL.16.ME/28.1**	Alicante	2016	Melon	4.30E-03	8.60E-03	5.60E-03	0	0	0	0
**B-AL.16.ZU/32.1**	Alicante	2016	Zucchini	6.20E-03	9.30E-03	1.21E-02	25	1	1.40E-03	8.85E-01
**B-MU.16.CU/34.1**	Murcia	2016	Cucumber	3.50E-03	1.26E-02	1.75E-02	38	2	1.90E-03	7.80E-01
**B-AL.16.CU/35.1**	Alicante	2016	Cucumber	3.70E-03	8.10E-03	1.58E-02	31	2	0	0
**B-MU.16.PU/37.1**	Alicante	2016	Pumpkin	3.30E-03	1.53E-02	1.20E-02	80	5	3.10E-03	8.19E-01
**B-MU.16.PU/38.1**	Alicante	2016	Pumpkin	3.60E-03	1.55E-02	1.18E-02	50	3	2.50E-03	7.84E-01
**B-MU.16.ZU/13.1**	Murcia	2016	Zucchini	3.90E-03	1.14E-02	7.60E-03	10	0	9.00E-04	5.57E-01
**B-MU.16.ZU/14.1**	Murcia	2016	Zucchini	3.30E-03	1.16E-02	2.55E-02	9	0	1.20E-03	9.06E-01
**B-MU.16.ZU/15.1**	Murcia	2016	Zucchini	2.60E-03	1.21E-02	1.31E-02	59	2	1.50E-03	8.16E-01
**B-MU.16.ZU/16.1**	Murcia	2016	Zucchini	3.20E-03	8.50E-03	9.70E-03	0	0	0	0
**B-LCM.16.PU/4.1**	La Mancha	2016	Melon	3.20E-03	4.60E-03	5.10E-03	0	0	0	0
**B-LCM.16.ME/5.1**	La Mancha	2016	Melon	2.80E-03	8.20E-03	8.10E-03	23	0	1.30E-03	9.20E-01
**B-LCM.16.ME/6.1**	La Mancha	2016	Melon	3.30E-03	1.13E-02	7.10E-03	32	1	1.00E-03	7.83E-01
**B-LCM.16.ME/1.1**	La Mancha	2016	Melon	3.10E-03	1.00E-02	5.60E-03	0	0	0	0
**B-LCM.16.ME/2.1**	La Mancha	2016	Melon	3.20E-03	4.30E-03	9.40E-03	26	1	1.20E-03	8.24E-01
**B-LCM.16.ME/3.1**	La Mancha	2016	Melon	3.50E-03	1.19E-02	1.16E-02	44	1	1.90E-03	8.26E-01


## Discussion

This study identifies natural hosts of ToLCNDV since its emergence in 2012 in cucurbit-producing areas of Spain, and also examines the genetic diversity and structure of ToLCNDV populations by performing deep full-genome sequencing analysis over different plant hosts, location and year. Our results show, firstly, that except for watermelon plants, ToLCNDV is widely distributed in cucurbit crops in Spain. Secondly, that *E. elaterium, D. stramonium*, and *S. oleraceus* are new and potential ToLCNDV weed hosts, and should be considered when trying to manage ToLCNDV diseases. Thirdly, that there is a monophyletic ToLCNDV population composed of isolates of the ES genotype, with no clustering pattern between plant hosts, location or year. And fourthly, that within-*Datura stramonium* populations exhibit high levels of genetic diversity that could shape the evolutionary dynamics of ToLCNDV populations in crops.

Spain is among the largest producer of cucurbits in the Mediterranean basin, mainly distributed over greenhouses as well as open-fields, accounting for a total of 62,500 ha cultivated in 2014 and corresponding to approximately 3 million tones harvested a year (MAPAMA, 2014). The use of genetic resistance to control viral diseases would be a desirable measure (Gómez et al., 2009a), but to date, only four accessions of *Cucurbita moschata* have been found to be symptomless or to display mild symptoms after ToLCNDV infection, appearing to be potential candidates as sources of ToLCNDV resistance (Sáez et al., 2016). When an integrated disease management program is attempted to help reduce the source of virus inoculum in the short-term, the knowledge about the cultivated and wild plant host range that can significantly affect disease incidence of a specific crop is highly relevant. In this sense, ToLCNDV has been described to affect a wide spectrum of plant species, such as aubergine, pepper, potato, tomato, lettuce, celery, bean and broad bean in the Indian subcontinent (Usharani et al., 2004; Pratap et al., 2011; Kushwaha et al., 2015). However, in the Mediterranean countries, ToLCNDV had been only identified infecting cucurbit and tomato plants (Juárez et al., 2014; Mnari-Hattab et al., 2015; Fortes et al., 2016; Panno et al., 2016; Yazdani-Khameneh et al., 2016; Parrella et al., 2017; Sifres et al., 2017; Zaidi et al., 2017), and its extent in further plant hosts was unknown. In this study, ToLCNDV appeared to infect cultivated cucurbit plants and tomato plants as well (see Table 1), although this was not the case in watermelon plants, being an interesting open question.

The recent biological characterization of a Spanish ToLCNDV isolate belonging to the ES type included watermelon, eggplant and pepper plants into the range of ToLCNDV plant hosts (Ruiz et al., 2017). A plausible explanation for the host range variation of ToLCNDV could be that the experimental inoculations in plants were carried out under laboratory conditions, while our plant surveys were focused on naturally symptomatic plants. It is likely that the severity of the plant symptoms may depend on the cultivar, the environment, the growing conditions, as well as the potential mixed infections with other plant viruses, and even the presence of betasatellites associated with ToLCNDV (Jyothsna et al., 2013). Thus, we attempted to detect the presence of other begomoviruses and/or betasatellites from our NGS data, as RCA amplification increases the chance of detecting any circular DNA, but after *de novo* assembling of sequence data from 80 libraries, neither other begomoviruses nor betasatellites were found. Nevertheless, it should be mentioned that tomato yellow leaf curl virus (TYLCV), which is a widely distributed virus of tomato, was also detected in four tomato samples in coinfections with ToLCNDV (D. Janssen, personal communication). This appears to be a matter of importance, as mixed infections of plant viruses are common in nature (Roossinck et al., 2010; Juárez et al., 2013), and their interactions within-plant could have far reaching consequences for the viral populations (Gómez et al., 2009b; Syller, 2011; Rodelo-Urrego et al., 2015), thus requiring further research.

Additionally, weed plants are also considered alternative hosts that may act as sources and reservoirs of begomovirus that could, afterward, infect nearby crop plants (Castillo-Urquiza et al., 2008; da Silva et al., 2011). In this sense, weed plants can greatly contribute to the prevalence and spatio-temporal distribution of viruses in crops (Ooi et al., 1997; Luis-Arteaga et al., 1998; García-Andrés et al., 2007; Kassem et al., 2013), and further increase diversity of begomoviruses (da Silva et al., 2011; Ferro et al., 2017). We found that *E. elaterium, D. stramonium, S. oleraceus*, and *S. nigrum* could be potential and wild alternative ToLCNDV hosts (see Table 1). Although experimental inoculations with a Spanish ToLCNDV isolate were negative for *S. nigrum* infection (Fortes et al., 2016), we found that 4 out of 11 *S. nigrum* plants were positive. Considering that these plants were derived from fields, other ecological factors may have favored virus infection in these natural conditions. Similarly, *D. stramonium* is another *Solanaceae* species that showed unequivocal virus-like symptoms (Figure 1) and was also positive for ToLCNDV. Note that *D. stramonium* is an annual herb that becomes a cosmopolitan weed in warm regions. Thus, after the evidence that begomoviruses can be transmitted from weed to cultivated-plant species (Castillo-Urquiza et al., 2008; Barreto et al., 2013), additional sampling is needed to conclude that *D. stramonium* may further increase ToLCNDV genetic diversity, although our results already highlight the importance of *D. stramonium* plants as inoculum sources and as reservoirs of viral diversity (see Figure 3 and Tables 2, 3). Nevertheless, it should be noted that further research fulfilling Koch’s postulates would provide a more accurately verification of these new hosts.

The population structure of ToLCNDV showed that Spanish isolates were uniformly distributed among the plant hosts surveyed in this study, as cucurbit isolates were clustered together with the isolates from other cultivated and wild plant hosts, and also showed the presence of genetic differentiation with isolates from the Indian subcontinent (see Figure 2). The molecular characterization of ToLCNDV based on full-genome sequencing was congruent and greatly supported the previous RFLP analysis of cucurbit-infecting ToLCNDV isolates from Spain (Fortes et al., 2016). This result may reflect specific features of ToLCNDV’s long- and short-dispersal. On the one hand, it could be speculated that the emergence of ToLCNDV in Spain came from the use of contaminated seeds, as seed transmission of other begomovirus (TYLCV) has been reported to occur in tomato plants (Kil et al., 2016), and it is well-known that global trade of seeds is greatly associated with the emergence and expansion of viral plant diseases (Hanssen et al., 2010). However, the same path that resulted in this long-range dispersal of the ToLCNDV-ES strain could be responsible for the introduction of other new variants, or these may already be present displacing the current viral strains, or even establishing recombination processes that generate some variants with selective advantage (Gómez et al., 2012; Lefeuvre and Moriones, 2015) shaping the evolutionary dynamics and epidemiology of this viral disease. On the other hand, ToLCNDV dispersal in Spain may also occur through contaminated seedlings, as cucurbit seedlings are produced in plant nurseries only located in the south of Spain. Thus, the occurrence and prevalence of ToLCNDV populations in central Spain (Castilla-La Mancha) could be due to the trade of contaminated seedlings. These data support the need of quarantine barriers, and highlight the importance of pathogen-free certifications to prevent the emergence of plant diseases as well as further epidemiological studies with continuous prospections in crops.

Begomoviruses have a high potential for increasing their genetic variability, as a result of high mutation and recombination rates, which are quite comparable to those reported for plant RNA viruses (Ge et al., 2007; Navas-Castillo et al., 2011; Lima et al., 2017). In particular, the presence of high intra-population diversity has been reported, which may allow for a rapid accumulation of variants during infection and generate diversity levels that allow for a rapid evolution and adaptation in response to new environments (Ge et al., 2007; Moriones and Navas-Castillo, 2008; Sánchez-Campos et al., 2018). For example, despite of invariant viral consensus sequences in TYLCV populations, the mutant swarm within-populations in cultivated tomato plants and wild hosts displayed a differential shape with greater complexity and heterogeneity in the alternative host, *S. nigrum* (Sánchez-Campos et al., 2018). In this study, and as mentioned above, within-host genetic diversity revealed that the level of diversity increased in 2016, and diversity of mutant spectra was higher in *Datura* sp. weed plants than in other plant species. Thus, it is possible that ToLCNDV host adaptation, agro-ecological factors or even genetic drift by vector transmission could lead to introgression or gene flow that disrupts the composition of the ToLCNDV population. A clear example for this potential disruption would be the well-known emergence of viruses associated with yellow curly tomato disease (Tomato yellow leaf curl disease, TYLCD). This disease was first described in Israel in 1929 associated with the increase in the population of its vector (also whitefly) and the disease spread to epidemic levels in tomato crops in a short-time (Moriones and Navas-Castillo, 2000; Díaz-Pendón et al., 2010; Lefeuvre et al., 2010).

Our findings suggest that wild plants could be key drivers for ToLCNDV genetic variability, and it could likely affect the genetic structure and spatio-temporal dynamics of the viral population. The potential plant virus adaptive evolution occurring in wild plants could be ecologically relevant to situations where emergent plant viruses have an advantage in agricultural systems. Therefore, there is a need for establishing continuous surveying and sampling protocols, including new geographic areas, which could allow us to have a broader epidemiological view of the disease, to know the structure and genetic variability of ToLCNDV in detail and to determine possible factors that favor the emergence of new viral variants in order to help the development of stable and effective strategies for the control of the disease.

## Author Contributions

MJ, MR, LM, and MT conducted the experiments. MR, LM, AG-P, and PG analyzed the data. MJ and PG wrote the manuscript. All authors read and approved the final manuscript.

## Conflict of Interest Statement

The authors declare that the research was conducted in the absence of any commercial or financial relationships that could be construed as a potential conflict of interest.

## Supplementary Material

TABLE S1 Number of ToLCNDV isolates that were randomly selected according to the plant host species, year and location in order to perform RCA and NGS sequencing analysis.

TABLE S2 Number of viral reads, mapped nucleotides and percentage identity with the reference genome for the 80 ToLCNDV consensus sequences.

TABLE S3 Statistic results for each PCA dimension from ToLCNDV-A genome, displaying the correlation coefficient and *p*-value for each variable analyzed.

TABLE S4 Statistic results for each PCA dimension from ToLCNDV-B genome, displaying the correlation coefficient and *p*-value for each variable analyzed.
